# A complete response to capecitabine and oxaliplatin chemotherapy in primary duodenal carcinoma with liver and nodal metastases: a case report

**DOI:** 10.1186/s40792-018-0532-2

**Published:** 2018-09-29

**Authors:** Seita Hagihara, Tetsunosuke Shimizu, Yoshihiro Inoue, Mitsuhiro Asakuma, Fumitoshi Hirokawa, Kohei Taniguchi, Michihiro Hayashi, Kazuhisa Uchiyama

**Affiliations:** Department of General and Gastroenterological Surgery, Osaka Medical College, 2-7 Daigaku machi, Takatsuki, Osaka, 569-8686 Japan

**Keywords:** Primary duodenal adenocarcinoma, Chemotherapy, Conversion surgery

## Abstract

**Background:**

Primary duodenal adenocarcinoma (PDC) is a rare and lethal disease, and cases with nodal or distant metastasis have a poor prognosis. There are several reports of unresectable duodenal adenocarcinoma responding to systemic chemotherapy. However, there is little data on conversion surgery for PDC with distant metastasis.

**Case presentation:**

We report a 55-year-old man with unresectable PDC with liver and nodal metastases responding to systemic chemotherapy with capecitabine and oxaliplatin (XELOX). His metastatic lesions completely disappeared by 18-fluorodeoxyglucose positron emission tomography/computed tomography after six courses of XELOX. Then, he underwent pancreaticoduodenectomy with lymph node dissection and partial resection of the liver. Postoperatively, the histological effect was determined to be grade 3, and the patient was diagnosed as having achieved pathological complete response (pCR). He is disease-free with no evidence of metastatic lesion for 14 months after surgery. Conversion surgery allowed R0 resection for unresectable PDC, and pCR can be achieved with XELOX treatment.

**Conclusion:**

To the best of our knowledge, this case is the first report of conversion surgery for unresectable PDC with liver and para-aortic lymph node metastases.

## Background

Primary duodenal adenocarcinoma (PDC) is a rare disease, and cases with nodal and distant metastases have a poor prognosis. Aggressive surgical resection for PDC is thought to be the most effective and only curative treatment. However, surgical removal of liver metastasis or para-aortic lymph node metastasis is still controversial. Moreover, guidelines for treatment have not been clearly defined because of the rarity of PDC. Therefore, clinicians must determine individual treatment plans.

Conversion surgery is a recent therapeutic option. Curative resection is performed after chemotherapy to remove tumors that were originally regarded as technically or oncologically unresectable or only marginally resectable [1].

Here, we report a very rare case of unresectable PDC with liver metastasis and para-aortic nodal metastasis that was successfully downgraded by systemic chemotherapy using six courses of capecitabine and oxaliplatin (XELOX) and conversion surgery.

## Case presentation

A 55-year-old man was referred to our hospital for jaundice and pruritus. His laboratory tests showed elevated blood markers, with a γ-glutamyl transpeptidase (γ-GTP) level of 1330 U/L (normal range, 10–75 U/L) and a total bilirubin level of 2.5 mg/dL (normal range, 0.5–2.5 mg/dL). Additionally, his serum carcinoembryonic antigen level was elevated at 17.4 U/mL (normal range, less than 5.0 U/mL), although there was no elevation in serum carbohydrate antigen 19-9 level.

**Fig. 1:**
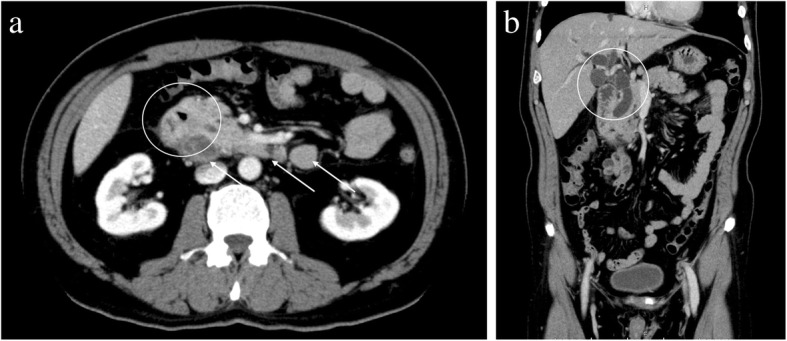
**a** The white circle shows wall thickening in the duodenum. The white arrows show the swelling of the para-aortic lymph nodes. **b** The white circle shows the dilatation of the bile duct

**Fig. 2:**
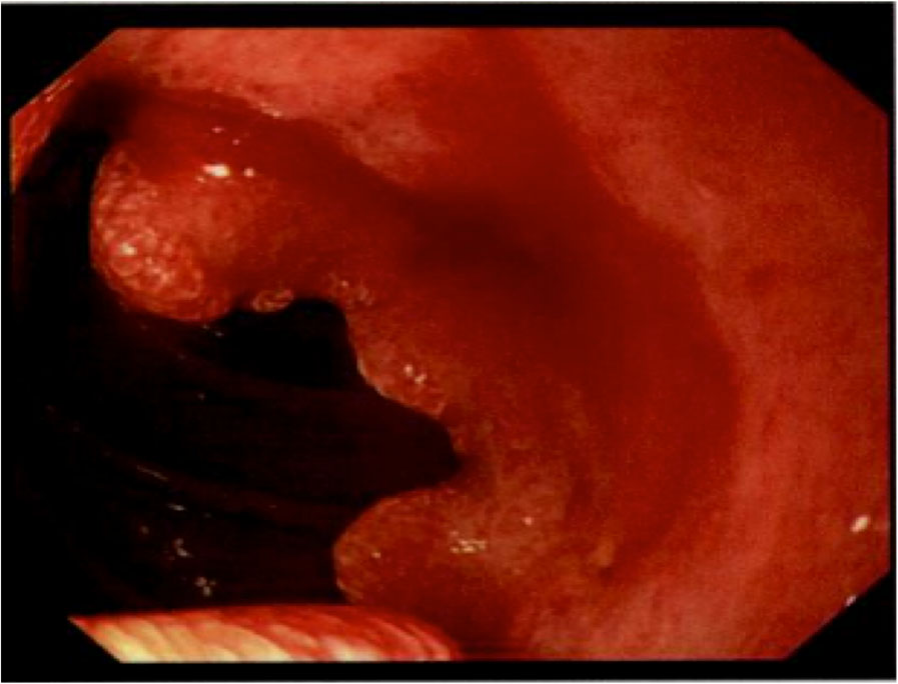
Upper endoscopy suggested a duodenal tumor

**Fig. 3:**
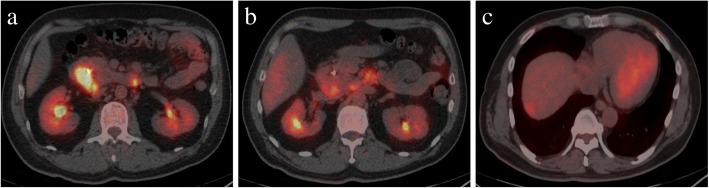
**a** Fluorodeoxyglucose positron emission tomography (FDG-PET) examination showed abnormal uptake in the tumor of the duodenum. **b** FDG-PET examination showed abnormal uptake in the para-aortic lymph nodes. **c** FDG-PET examination showed abnormal uptake in segment 8 of the liver

Computed tomography (CT) showed wall thickening in the second portion of the duodenum, dilation of the common bile duct, and swelling of the para-aortic lymph node (Fig. 1). Upper endoscopy suggested a duodenal tumor (Fig. 2). Although intraductal ultrasonography was performed, invasion of the tumor into the bile duct was not observed. For obstructive jaundice, an endoscopic retrograde bile drainage tube was placed at the common bile duct. Further imaging with 18-fluorodeoxyglucose positron emission tomography (FDG-PET)/CT demonstrated abnormal uptake in the tumor in the duodenum (Fig. 3a), in the para-aortic lymph nodes (Fig. 3b), and in a 10-mm metastasis in segment 8 of the liver (Fig. 3c). Based on the Union for International Cancer Control TMN staging, the clinical diagnosis was cT4N2M1, stage IV.

The patient was scheduled for combined chemotherapy with XELOX: 2000 mg/m^2^ capecitabine orally on days 1–15 and 130 mg/m^2^ oxaliplatin intravenously on day 1 of a 21-day cycle. The patient received six cycles and experienced no adverse events.

**Fig. 4:**
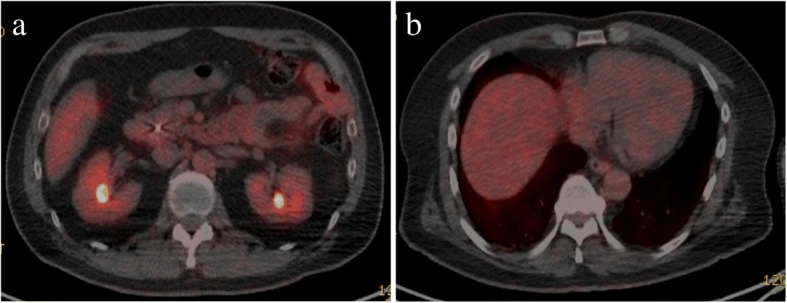
**a** Fluorodeoxyglucose positron emission tomography (FDG-PET) after chemotherapy showed no uptake in the tumor in the duodenum or the para-aortic lymph nodes. **b** FDG-PET after chemotherapy showed no uptake in segment 8 of the liver

In order to address the effectiveness of chemotherapy, the patient received follow-up FDG-PET/CT or CT every 2 months. Last FDG-PET/CT confirmed disappearance of the metastatic liver tumor and nodal metastasis (Fig. 4a, b). The patient was considered a surgical candidate to be evaluated for complete response (CR) by FDG-PET/CT. Seven months after the initial diagnosis and after 6 months of chemotherapy, the patient underwent pancreaticoduodenectomy with lymph node dissection and partial resection of the liver for curative intent.

**Fig. 5:**
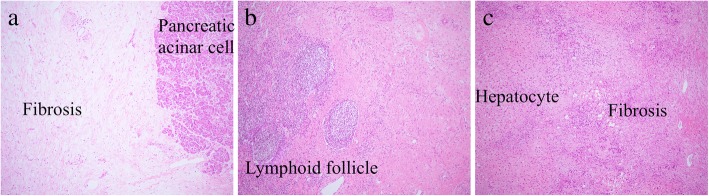
Histology of duodenum (**a**), lymph node (**b**), and hepatic (**c**) specimens following resection (hematoxylin-eosin staining, × 10). No residual carcinoma in found on histologic examination. All margins are negative for cancer on histologic examination

During the operation, peritoneal dissemination and ascites were not observed. D2 lymph node dissection, including resection of the para-aortic lymph node, which is based on the dissection range of pancreatic cancer, was performed. Regarding liver metastasis, we could detect it as a scar. The lesions in the duodenum, liver, and 43 lymph nodes were not visible in the surgical specimen. Postoperatively, the histological effect was determined to be grade 3, and the patient was diagnosed as having achieved pathological CR (Fig. 5a, b, c).

Although the patient suffered from grade A postoperative pancreatic fistula based on the International Study Group of Pancreatic Fistula definition, he was discharged from our hospital on postoperative day 32 without fatal complications. He received capecitabine orally for 6 months and has been disease-free for 14 months after the surgery with no evidence of metastatic lesion.

### Discussion

PDC is a very rare disease; the cancer accounts for less than 0.3–1% of all digestive organ cancers [2] and 25–45% of primary small intestinal cancers. Aggressive surgical resection for PDC is thought to be the most effective treatment and only chance for a cure. The 5-year survival rate for patients who undergo curative resection is 50–70%. However, patients who present with unresectable disease receive palliative operation or endoscopic treatment with or without chemotherapy, and their prognosis remains poor, despite considerable research.

In recent years, advancements in chemotherapy have allowed conversion surgery to emerge as a new therapeutic option. It is expected to have beneficial effects for various cancers, including pancreatic cancer [3] and gastric cancer [4]. However, there are very few reports of conversion surgery for unresectable PDC. Kanehira et al. reported a conversion surgery in which they achieved R0 resection for PDC with para-aortic lymph node metastasis using S-1 and cisplatin combination chemotherapy [5]. However, even the feasibility of chemotherapy for PDC is unclear. Also, the most effective regimens are controversial. Treatment with S-1 and cisplatin, which is a standard treatment for gastric cancer, and FOLFILI or FOLFOX, standard treatments for colon cancer, have been used [8, 9]. Xian et al. reported that a modified FOLFOX regimen, which consists of biweekly oxaliplatin in combination with continuous infusional 5-fluorouracil and leucovorin, had efficacy and safety in 33 patients with unresectable small bowel cancer, including 26 patients with duodenal cancer. They observed only one CR, and the 15 patients who achieved partial response received a median of nine cycles [10]. Although Tsushima et al. and Zaanan et al. recommended a platinum-based regimen, such as FOLFOX, for unresectable intestinal cancer [11, 12], none of the patients in these studies achieved CR, despite platinum-based regimens being common in unresectable PDC.

Our patient received XELOX with the aim of eliminating the liver and lymph node metastases or decreasing them to operable sizes. Fortunately, our patient was able to achieve pathological complete response (pCR). This case also demonstrates two important clinical issues. First, this combination therapy, which is a platinum-based regimen, is safe and effective for patients with colon cancer. Second, the tumor, including the metastatic lesions, disappeared rapidly without severe toxicity. Moreover, PET/CT was beneficial for therapeutic evaluation in our case, as has been previously shown by several investigators [13].

Conversion surgery for PDC has some limitations. Even retrospective data are limited by the accuracy and completeness of the medical record, and the case studies reported in many previous studies have been collected over several decades. Therefore, there is no clear evidence that conversion surgery improves outcomes in patients with unresectable PDC.

Moreover, there is no clear evidence for dissection of para-aortic lymph node metastasis from digestive cancer including PDC. In 2014, Tsubraya reported the effectiveness of para-aortic lymph node dissection for gastric cancer with extensive lymph node metastasis including para-aortic lymph node metastasis after neoadjuvant chemotherapy [6]. Also, Arimoto reported that the 3-year overall survival was 41.2%, though the recurrence rate after para-aortic lymph node dissection for patients with colorectal cancer receiving neoadjuvant or adjuvant chemotherapy was quite high [7]. So, we considered that there may be a value in trying para-aortic lymph node dissection in such our case.

In terms of watch and wait strategy, it is the agenda that is needed to debate for patients who achieved complete response. Renehan et al. reported the effectiveness of the wait and watch strategy for rectal cancer. In fact, 3-year non-regrowth free survival and 3-year overall survival rate for patients who received wait and watch approach did not show no significant difference compared with patients who underwent surgical treatment. Moreover, patients who were managed by watch and wait avoided major surgery and averted permanent colostomy without loss of oncological safety at 3 years [14]. However, in terms of watch and wait approach for patients with duodenal carcinoma who achieved complete response, none of the reports has been published. So, we should carefully determine the surgical indication for those patients, and it is needed to debate this agenda with those accumulating cases.

## Conclusion

Here, we reported a case of pCR in a patient with metastatic PDC. To the best of our knowledge, this case is the first report of conversion surgery for unresectable PDC with liver and para-aortic lymph node metastases. XELOX seemed to be effective and useful as a regimen of chemotherapy, but several issues remain to be resolved with respect to chemotherapy for PDC, such as selection of the patient and assessment method of therapeutic effects in comparison with other regimens. Therefore, we need additional cases to be evaluated to determine whether this is a suitable therapeutic option in the fight against highly aggressive cancer.

## Abbreviations

FDG-PET: 18-fluorodeoxyglucose positron emission tomography
pCR: Pathological complete response
PDC: Primary duodenal adenocarcinoma
